# Supplementary material for: A Likelihood Approach for Real-Time Calibration of Stochastic Compartmental Epidemic Models
Source: PLoS Comput Biol. 2017 Jan 17;13(1):e1005257. doi: 10.1371/journal.pcbi.1005257 (PMC5240920; doi:10.1371/journal.pcbi.1005257)
Supplement: S1 File — (TAR.GZ) [file pcbi.1005257.s014.tar.gz › HSPH_Online-SI-Revision/Additions/PF09/n100-extreme-SEITR_noisy/n100-extreme-SEITR_noisy_PF_table0.pdf]

1	R0	{1.38654, 9.85565}	{2.06465, 20.}	{{2.06312, 20.}, {2.06568, 20.}}	{48.9063, 102.929}	inf	5261.	13 244.2	{13 215.7, 13 270.4}	28.9971	0.148724	newly	12 047.4	4830.97	{4745., 4913.}	59.9002	0.396898	Reff	0.312221	1.27903	{1.27842, 1.28}	309.657	ar	92 379.	87 396.1	{87 020., 87 731.}	5.39393
2	R0	{2.36629, 12.2029}	{3., 20.}	{{3., 20.}, {3., 20.}}	{26.7807, 63.8955}	inf	4672.	30 486.2	{30 380.1, 30 589.7}	12.9166	0.0600666	newly	17 453.	11 224.9	{11 110., 11 353.}	35.6848	0.191702	Reff	0.202507	1.43818	{1.43539, 1.44093}	610.186	ar	99 022.	101 331.	{100 978., 101 651.}	2.33202
3	R0	{2.32253, 12.836}	{3., 20.}	{{3., 20.}, {3., 20.}}	{29.1695, 55.8118}	inf	6387.	28 285.4	{28 148.8, 28 398.7}	22.0746	0.108324	newly	18 266.	10 640.6	{10 520., 10 759.}	41.7462	0.234695	Reff	0.29092	1.56245	{1.56001, 1.566}	437.071	ar	98 997.	100 682.	{100 278., 101 023.}	1.70174
4	R0	{1.79075, 11.2058}	{3., 20.}	{{3., 20.}, {3., 20.}}	{67.5276, 78.479}	inf	6785.	32 736.6	{32 342.2, 33 098.5}	16.9666	0.0680457	newly	16 022.	11 555.6	{11 378., 11 705.}	27.877	0.141953	Reff	0.341174	1.17541	{1.16851, 1.18106}	244.519	ar	96 920.	103 575.	{102 932., 104 376.}	6.86698
5	R0	{2.26952, 9.62371}	{3., 20.}	{{3., 20.}, {3., 20.}}	{32.1865, 107.82}	inf	2810.	28 059.	{27 942.3, 28 154.9}	4.73616	0.0210738	newly	16 920.	10 640.7	{10 513., 10 777.}	37.1118	0.201448	Reff	0.133902	1.61571	{1.61291, 1.61801}	1106.64	ar	98 881.	101 680.	{101 333., 102 018.}	2.8308
6	R0	{1.57191, 6.89004}	{3., 20.}	{{3., 20.}, {3., 20.}}	{90.8506, 190.274}	inf	4582.	31 703.7	{31 506.5, 31 906.3}	50.3612	0.17713	newly	17 405.	11 485.	{11 325., 11 623.}	34.0132	0.180564	Reff	0.240125	1.33727	{1.33407, 1.34117}	456.905	ar	95 046.	103 878.	{103 389., 104 328.}	9.29221
7	R0	{1.99226, 17.2339}	{2.12442, 20.}	{{2.12049, 20.}, {2.12736, 20.}}	{6.63356, 16.0503}	inf	5638.	17 492.4	{17 429.5, 17 561.}	41.7366	0.234607	newly	11 332.	6320.37	{6224., 6407.}	44.2255	0.25359	Reff	0.270071	1.2349	{1.23283, 1.23682}	357.249	ar	97 992.	90 068.5	{89 713., 90 394.}	8.08585
8	R0	{2.63369, 15.1879}	{3., 20.}	{{3., 20.}, {3., 20.}}	{13.9086, 31.6838}	inf	3741.	31 281.7	{31 193., 31 364.4}	15.0047	0.0706062	newly	14 766.	11 282.3	{11 140., 11 411.}	23.5926	0.11688	Reff	0.159786	1.31139	{1.30973, 1.31326}	720.714	ar	99 451.	101 284.	{100 960., 101 550.}	1.84339
9	R0	{2.53271, 9.90552}	{3., 20.}	{{3., 20.}, {3., 20.}}	{18.4502, 101.908}	inf	2374.	23 059.5	{22 955.1, 23 162.7}	26.4707	0.133543	newly	17 411.	9282.25	{9173., 9404.}	46.6874	0.273193	Reff	0.11382	1.94516	{1.94116, 1.94846}	1608.98	ar	99 355.	101 589.	{101 290., 101 907.}	2.24841
10	R0	{2.48791, 18.9003}	{2.79632, 20.}	{{2.78283, 20.}, {2.84215, 20.}}	{12.3965, 5.81843}	inf	6612.	27 564.3	{27 468.9, 27 708.3}	28.3504	0.144789	newly	13 614.	9881.85	{9764., 9989.}	27.4141	0.139164	Reff	0.296509	1.24811	{1.24637, 1.25037}	320.936	ar	99 291.	97 496.7	{97 033., 98 911.}	1.80709
11	R0	{2.84414, 18.4765}	{2.2666, 20.}	{{2.26118, 20.}, {2.26977, 20.}}	{20.3063, 8.24561}	inf	5335.	14 103.7	{14 000.9, 14 179.4}	66.3765	0.473363	newly	14 675.	5508.87	{5398., 5593.}	62.4609	0.425552	Reff	0.221814	1.68113	{1.67871, 1.68271}	657.9	ar	99 654.	92 881.4	{92 535., 93 191.}	6.7961
12	R0	{2.29369, 14.622}	{2.91998, 20.}	{{2.91596, 20.}, {2.92357, 20.}}	{27.3049, 36.7802}	inf	4308.	28 678.6	{28 620.3, 28 726.4}	13.7355	0.0641692	newly	14 159.	10 231.6	{10 121., 10 317.}	27.738	0.141105	Reff	0.188656	1.2257	{1.22427, 1.22684}	549.7	ar	98 982.	98 785.	{98 643., 98 966.}	0.201552
13	R0	{2.73413, 16.7529}	{2.80453, 20.}	{{2.80302, 20.}, {2.80657, 20.}}	{2.57474, 19.3823}	inf	4068.	25 543.1	{25 496.4, 25 596.7}	34.6004	0.184426	newly	14 704.	9504.34	{9391., 9627.}	35.3622	0.189533	Reff	0.173699	1.47389	{1.47325, 1.47459}	748.532	ar	99 543.	97 193.7	{97 022., 97 353.}	2.36012
14	R0	{1.79554, 16.5943}	{1.93823, 20.}	{{1.93592, 20.}, {1.94205, 20.}}	{7.94702, 20.5233}	inf	7555.	11 907.2	{11 868.5, 11 972.}	57.8343	0.375052	newly	11 642.	4420.57	{4326., 4502.}	62.0291	0.420595	Reff	0.390135	1.38367	{1.38278, 1.38505}	254.665	ar	96 821.	83 540.4	{83 133., 83 938.}	13.7167
15	R0	{1.91429, 13.7771}	{1.91902, 20.}	{{1.91801, 20.}, {1.92252, 20.}}	{0.389842, 45.1684}	inf	5519.	9650.13	{9641.49, 9691.41}	66.3054	0.472458	newly	13 230.	3664.95	{3584., 3747.}	72.2982	0.557578	Reff	0.264038	1.49499	{1.49468, 1.49692}	466.202	ar	97 541.	82 610.6	{82 103., 83 084.}	15.3068
16	R0	{1.42094, 10.5871}	{2.16029, 17.2263}	{{2.15754, 17.1946}, {2.16235, 17.2613}}	{52.0327, 62.7104}	inf	7009.	17 274.6	{17 200.3, 17 323.6}	15.2541	0.0718829	newly	12 953.	7339.98	{7244., 7435.}	43.3337	0.246699	Reff	0.417131	1.28487	{1.2827, 1.28735}	208.025	ar	93 044.	87 851.8	{87 525., 88 217.}	5.58037
17	R0	{1.45515, 6.16466}	{3., 20.}	{{3., 20.}, {3., 20.}}	{106.164, 224.43}	inf	6217.	28 816.	{28 669.1, 28 935.5}	38.926	0.142781	newly	20 574.	10 933.2	{10 811., 11 045.}	46.859	0.274586	Reff	0.356002	1.62193	{1.61716, 1.62557}	355.595	ar	93 254.	103 893.	{103 422., 104 242.}	11.4083
18	R0	{2.85669, 13.2416}	{3., 20.}	{{3., 20.}, {3., 20.}}	{5.01665, 51.0391}	inf	3137.	27 280.2	{27 175.3, 27 375.8}	28.8448	0.147795	newly	17 189.	10 399.	{10 271., 10 523.}	39.5021	0.218277	Reff	0.132179	1.63613	{1.63374, 1.63907}	1137.81	ar	99 644.	101 663.	{101 255., 101 953.}	2.02662
19	R0	{2.56417, 17.9558}	{2.24003, 20.}	{{2.23867, 20.}, {2.24113, 20.}}	{12.6411, 11.3846}	inf	6398.	15 222.6	{15 182.9, 15 259.4}	61.5329	0.414912	newly	14 542.	5844.3	{5760., 5927.}	59.8109	0.395916	Reff	0.282084	1.58559	{1.58461, 1.58698}	462.097	ar	99 389.	91 690.7	{91 406., 91 952.}	7.74562
20	R0	{2.27517, 11.1864}	{3., 20.}	{{3., 20.}, {3., 20.}}	{31.8583, 78.7885}	inf	3799.	31 539.6	{31 361.7, 31 712.2}	1.32151	0.00578131	newly	16 864.	11 468.1	{11 336., 11 614.}	31.9963	0.167487	Reff	0.172117	1.36216	{1.35751, 1.36682}	691.42	ar	98 894.	104 130.	{103 619., 104 560.}	5.29413
21	R0	{2.35735, 10.4735}	{3., 20.}	{{3., 20.}, {3., 20.}}	{27.2615, 90.9581}	inf	3960.	27 546.5	{27 434.3, 27 636.2}	17.4785	0.0834347	newly	18 543.	10 571.5	{10 440., 10 685.}	42.9892	0.24406	Reff	0.172039	1.6862	{1.68308, 1.68881}	880.122	ar	99 036.	102 724.	{102 191., 103 092.}	3.72408
22	R0	{1.79551, 11.9179}	{2.9998, 20.}	{{3., 20.}, {3., 20.}}	{67.0722, 67.8148}	inf	5574.	31 258.6	{30 982.8, 31 486.}	16.9375	0.0679422	newly	14 003.	10 839.4	{10 679., 10 995.}	22.5921	0.111243	Reff	0.270045	1.05628	{1.05359, 1.05826}	291.148	ar	97 033.	100 803.	{100 221., 101 529.}	3.88517
23	R0	{1.23545, 6.31687}	{3., 20.}	{{3., 20.}, {3., 20.}}	{142.827, 216.612}	inf	4400.	32 017.5	{31 922.8, 32 100.5}	129.632	0.361031	newly	13 260.	10 908.9	{10 791., 11 006.}	17.7308	0.084775	Reff	0.306775	0.941738	{0.939204, 0.94491}	206.981	ar	88 739.	99 924.4	{99 706., 100 171.}	12.6048
24	R0	{2.24089, 10.1789}	{3., 20.}	{{3., 20.}, {3., 20.}}	{33.8754, 96.4849}	inf	5491.	25 218.2	{25 118.4, 25 290.5}	25.3501	0.126972	newly	20 012.	9902.76	{9772., 10 011.}	50.5159	0.305555	Reff	0.242442	1.80975	{1.80755, 1.81206}	646.466	ar	98 868.	100 902.	{100 536., 101 179.}	2.05747
25	R0	{1.57298, 10.2607}	{2.17243, 16.6191}	{{2.16765, 16.5901}, {2.17424, 16.6616}}	{38.109, 61.9682}	inf	7361.	15 554.1	{15 471.5, 15 601.9}	34.6605	0.184827	newly	15 457.	7075.03	{6977., 7184.}	54.2277	0.339426	Reff	0.403627	1.43231	{1.43027, 1.43467}	254.86	ar	94 957.	87 418.7	{87 124., 87 668.}	7.93861
26	R0	{1.2154, 8.2576}	{2.15682, 16.9905}	{{2.15492, 16.9486}, {2.15873, 17.0581}}	{77.4577, 105.756}	inf	5404.	18 623.2	{18 572., 18 660.2}	23.4957	0.0916508	newly	11 730.	7797.51	{7701., 7904.}	33.5251	0.177365	Reff	0.35796	1.1361	{1.13307, 1.13963}	217.381	ar	88 421.	88 916.8	{88 656., 89 170.}	0.56076
27	R0	{2.22841, 18.7983}	{2.17612, 17.0406}	{{2.17169, 16.9857}, {2.17834, 17.0909}}	{2.34633, 9.35058}	inf	5503.	19 147.3	{19 078.7, 19 197.8}	43.7787	0.2501	newly	11 908.	8056.54	{7947., 8149.}	32.3435	0.169712	Reff	0.242317	1.18397	{1.18026, 1.18788}	388.602	ar	98 756.	91 776.7	{91 514., 92 037.}	7.06719
28	R0	{1.27778, 5.36767}	{3., 20.}	{{3., 20.}, {3., 20.}}	{134.782, 272.601}	inf	5294.	30 057.8	{29 907.2, 30 183.5}	81.9262	0.259893	newly	18 063.	11 074.1	{10 935., 11 203.}	38.6917	0.212499	Reff	0.340107	1.4374	{1.43466, 1.4408}	322.633	ar	89 843.	101 404.	{101 008., 101 722.}	12.8682
29	R0	{2.38042, 15.8901}	{3., 20.}	{{3., 20.}, {3., 20.}}	{26.0282, 25.8645}	inf	6676.	29 781.6	{29 673.4, 29 864.9}	19.9657	0.0967251	newly	15 388.	10 787.2	{10 663., 10 889.}	29.8986	0.154287	Reff	0.307407	1.32809	{1.325, 1.33135}	332.028	ar	99 112.	97 954.4	{97 770., 98 145.}	1.16799
30	R0	{2.53188, 12.7607}	{3., 20.}	{{3., 20.}, {3., 20.}}	{18.489, 56.7312}	inf	7079.	25 605.5	{25 491.7, 25 709.1}	34.7164	0.185198	newly	19 690.	10 077.6	{9964., 10 211.}	48.8185	0.290906	Reff	0.319143	1.82523	{1.82223, 1.82787}	471.916	ar	99 317.	102 995.	{102 518., 103 425.}	3.70309
31	R0	{1.24482, 10.5471}	{2.28835, 20.}	{{2.28674, 20.}, {2.28931, 20.}}	{83.8296, 89.6256}	inf	5495.	22 250.2	{22 183.4, 22 299.1}	40.9311	0.149006	newly	9931.	7639.78	{7534., 7743.}	23.0714	0.113934	Reff	0.364384	0.93949	{0.937725, 0.941608}	157.83	ar	89 097.	96 402.9	{96 100., 96 730.}	8.19995
32	R0	{2.30446, 17.9607}	{3., 20.}	{{3., 20.}, {3., 20.}}	{30.1823, 11.3542}	inf	6256.	31 422.3	{31 308., 31 517.8}	12.1202	0.0561124	newly	13 001.	10 764.6	{10 650., 10 888.}	17.2015	0.0819936	Reff	0.270705	0.983747	{0.977955, 0.9912}	263.402	ar	99 028.	98 422.6	{98 173., 98 615.}	0.611393
33	R0	{2.31579, 11.8236}	{3., 20.}	{{3., 20.}, {3., 20.}}	{29.5454, 69.1532}	inf	3005.	33 046.8	{32 810.3, 33 223.2}	8.83889	0.0367796	newly	15 166.	11 774.3	{11 649., 11 898.}	22.3637	0.109951	Reff	0.14124	1.2384	{1.23477, 1.2424}	776.804	ar	99 012.	105 275.	{104 724., 105 629.}	6.32598
34	R0	{1.6678, 10.6744}	{3., 20.}	{{3., 20.}, {3., 20.}}	{79.8777, 87.3642}	inf	4965.	31 325.1	{31 237.7, 31 389.1}	34.1431	0.127568	newly	13 645.	10 605.5	{10 518., 10 716.}	22.2755	0.109454	Reff	0.259643	0.901995	{0.899049, 0.905488}	247.398	ar	95 809.	99 002.7	{98 800., 99 178.}	3.33343
35	R0	{1.65476, 7.07559}	{3., 20.}	{{3., 20.}, {3., 20.}}	{81.2952, 182.662}	inf	5229.	27 866.2	{27 730.2, 27 957.3}	18.3327	0.0731027	newly	19 217.	10 628.	{10 519., 10 715.}	44.6947	0.257246	Reff	0.270404	1.64636	{1.64333, 1.64987}	508.851	ar	95 746.	102 778.	{102 319., 103 193.}	7.34493
36	R0	{2.71807, 18.5959}	{2.94452, 20.}	{{2.93527, 20.}, {2.95074, 20.}}	{8.33115, 7.55059}	inf	7257.	29 038.1	{28 844.7, 29 189.8}	30.6372	0.15888	newly	15 024.	10 631.7	{10 490., 10 764.}	29.2354	0.150204	Reff	0.322961	1.3943	{1.39236, 1.39696}	331.724	ar	99 540.	99 651.7	{99 322., 99 936.}	0.230812
37	R0	{2.6202, 12.7552}	{2.86377, 20.}	{{2.85875, 20.}, {2.86644, 20.}}	{9.29576, 56.7988}	inf	5145.	21 005.6	{20 899.1, 21 075.9}	46.3199	0.270189	newly	18 951.	8392.51	{8294., 8505.}	55.7147	0.353762	Reff	0.212734	1.87998	{1.87803, 1.88128}	783.724	ar	99 426.	98 904.	{98 631., 99 151.}	0.525014
38	R0	{1.47672, 9.81651}	{2.13826, 16.5332}	{{2.13659, 16.5271}, {2.13874, 16.5834}}	{44.7982, 68.4227}	inf	5442.	17 031.2	{16 978.5, 17 070.}	18.2999	0.0877783	newly	13 535.	7528.94	{7400., 7632.}	44.3743	0.254758	Reff	0.299449	1.26349	{1.26172, 1.26575}	321.937	ar	93 815.	87 162.4	{86 859., 87 476.}	7.09118
39	R0	{1.90004, 12.3313}	{2.8753, 20.}	{{2.872, 20.}, {2.87865, 20.}}	{51.3283, 62.1889}	inf	6237.	28 489.7	{28 414.4, 28 549.8}	0.926119	0.00404151	newly	15 042.	10 125.7	{10 019., 10 243.}	32.6842	0.171899	Reff	0.303056	1.19715	{1.19531, 1.19962}	295.026	ar	97 413.	99 061.3	{98 875., 99 261.}	1.69206
40	R0	{2.64218, 18.5237}	{3., 20.}	{{3., 20.}, {3., 20.}}	{13.5426, 7.96979}	inf	4328.	31 195.2	{31 102.8, 31 269.6}	17.6148	0.0841518	newly	13 194.	10 655.5	{10 535., 10 771.}	19.2397	0.0928157	Reff	0.17626	0.962929	{0.960266, 0.966489}	446.312	ar	99 474.	100 396.	{100 148., 100 606.}	0.926674
41	R0	{1.6945, 7.17996}	{3., 20.}	{{3., 20.}, {3., 20.}}	{77.0434, 178.553}	inf	7462.	24 387.	{24 303.5, 24 447.1}	7.80294	0.0352843	newly	23 349.	9629.62	{9523., 9731.}	58.7579	0.384677	Reff	0.379415	1.83892	{1.83536, 1.84107}	384.672	ar	96 332.	101 305.	{100 976., 101 569.}	5.162
42	R0	{1.84559, 18.0074}	{2.27883, 20.}	{{2.26815, 20.}, {2.28754, 20.}}	{23.4741, 11.0655}	inf	6919.	21 769.2	{21 637.8, 21 898.8}	24.2574	0.120665	newly	10 541.	7654.53	{7552., 7755.}	27.3833	0.138983	Reff	0.338389	1.08843	{1.08522, 1.09155}	221.651	ar	97 358.	95 947.4	{95 178., 96 628.}	1.44885
43	R0	{2.24965, 17.0361}	{2.8836, 20.}	{{2.87855, 20.}, {2.88866, 20.}}	{28.1799, 17.3978}	inf	7490.	28 477.3	{28 394.3, 28 546.}	19.3551	0.0934243	newly	14 237.	10 127.8	{10 014., 10 240.}	28.8628	0.147919	Reff	0.339337	1.20504	{1.20095, 1.21185}	255.116	ar	98 770.	99 327.4	{99 146., 99 480.}	0.564362
44	R0	{2.54324, 15.7967}	{3., 20.}	{{3., 20.}, {3., 20.}}	{17.9596, 26.6087}	inf	3895.	31 092.6	{30 953.3, 31 160.1}	14.2251	0.0666409	newly	14 170.	11 025.3	{10 920., 11 149.}	22.1929	0.108996	Reff	0.167193	1.19882	{1.19747, 1.20069}	617.029	ar	99 354.	99 845.2	{99 567., 100 112.}	0.494384
45	R0	{2.54943, 14.8032}	{3., 20.}	{{3., 20.}, {3., 20.}}	{17.6734, 35.1059}	inf	4134.	31 465.	{31 340.8, 31 556.5}	13.2671	0.0618174	newly	14 996.	11 298.1	{11 178., 11 431.}	24.6591	0.122984	Reff	0.179352	1.28584	{1.28345, 1.28861}	616.933	ar	99 380.	101 078.	{100 660., 101 437.}	1.70827
46	R0	{2.37755, 12.6461}	{3., 20.}	{{3., 20.}, {3., 20.}}	{26.1803, 58.1515}	inf	5896.	28 487.1	{28 363.3, 28 580.}	21.4302	0.104746	newly	17 991.	10 691.4	{10 566., 10 817.}	40.5736	0.226038	Reff	0.261673	1.54894	{1.54596, 1.55171}	491.938	ar	99 096.	100 353.	{100 033., 100 646.}	1.2689
47	R0	{1.69279, 11.6636}	{2.23676, 17.562}	{{2.22633, 17.5337}, {2.24405, 17.5956}}	{32.1343, 50.5713}	inf	6849.	16 132.1	{15 984.5, 16 237.}	37.4434	0.203736	newly	14 547.	6991.98	{6876., 7088.}	51.9352	0.318205	Reff	0.3499	1.48596	{1.4827, 1.48865}	324.681	ar	96 214.	90 033.1	{89 720., 90 280.}	6.42407
48	R0	{1.82402, 10.0038}	{3., 20.}	{{3., 20.}, {3., 20.}}	{64.4719, 99.924}	inf	6918.	30 584.2	{30 414.6, 30 718.3}	9.54238	0.0395794	newly	17 877.	11 206.7	{11 069., 11 336.}	37.3123	0.202837	Reff	0.343281	1.41361	{1.41108, 1.41621}	311.795	ar	97 076.	101 845.	{101 551., 102 160.}	4.91261
49	R0	{1.6424, 11.1667}	{3., 20.}	{{3., 20.}, {3., 20.}}	{82.6595, 79.1039}	inf	4677.	31 345.9	{31 236.3, 31 445.9}	38.6802	0.142013	newly	12 499.	10 425.	{10 286., 10 559.}	16.5932	0.0788213	Reff	0.249793	0.784191	{0.778476, 0.790832}	213.937	ar	95 546.	100 340.	{99 989., 100 638.}	5.017
50	R0	{2.05638, 14.3048}	{3., 20.}	{{3., 20.}, {3., 20.}}	{45.8874, 39.8132}	inf	6276.	32 268.2	{32 144.8, 32 391.1}	2.14044	0.00919564	newly	14 245.	11 137.2	{10 991., 11 261.}	21.8167	0.106905	Reff	0.290299	1.03065	{1.02523, 1.03787}	255.031	ar	98 194.	100 607.	{100 276., 100 893.}	2.45728
